# Factors affecting availability for detection: An example using radio-collared Northern Bobwhite (*Colinus virginianus*)

**DOI:** 10.1371/journal.pone.0190376

**Published:** 2017-12-22

**Authors:** Christopher M. Lituma, David A. Buehler, Evan P. Tanner, Ashley M. Tanner, Patrick D. Keyser, Craig A. Harper

**Affiliations:** Department of Forestry, Wildlife and Fisheries, University of Tennessee, Knoxville, Tennessee, United States of America; Hungarian Academy of Sciences, HUNGARY

## Abstract

Avian monitoring strategies are usually linked to bird singing or calling behavior. Individual availability for detection can change as a result of conspecific factors affecting bird behavior, though the magnitude of these effects is difficult to quantify. We evaluated behavioral and temporal factors affecting Northern Bobwhite (*Colinus virginianus*) breeding season individual availability for detection during three common survey times (3 min, 5 min, 10 min). We conducted 10-minute surveys associated with radio-collared male Northern Bobwhites on Peabody Wildlife Management Area, Kentucky, from 2010–2011. We homed to within 50 m of radio-collared males and recorded number of distinct Northern Bobwhite whistles (singing rate) per 1-minute interval, number of other males calling during the survey, minutes-since-sunrise, and day-of-season. We also recorded the number of minutes during a 10-minute survey that radio-collared male Northern Bobwhites called. We used logistic regression to estimate availability of radio-collared individuals for 3-minute, 5-minute, and 10-minute surveys. We also modeled number of minutes during 10-minute surveys that radio-collared Northern Bobwhites called, and we modeled singing rate. Individual availability for detection of radio-collared individuals during a 10-minute survey increased by 100% when at least 1 other Northern Bobwhite called during a survey (6.5% to 13.1%) and by 626% when 6 other Northern Bobwhites were calling (6.5% to 47.6%). Individual availability was 30% greater for 10-minute surveys than 5-minute surveys or 55% greater for 10-minute surveys than 3-minute surveys. Northern Bobwhite called most (2.8 ± 0.66 minutes/10-min survey) and at a greater rate (11.8 ± 1.3 calls/10-min period) when at least 5 other Northern Bobwhites called. Practitioners risk biasing population estimates low if individual availability is unaccounted for because species with low populations will not be stimulated by other calling males, are less likely to call, call less frequently, and call fewer times per minute, reducing their individual availability and likelihood to be counted on a survey even when they are present.

## Introduction

Bird singing is a behavior used by many species to attract a mate [1], alert competitors to territorial boundaries [2], or to prevent extra pair copulations [3]. Bird songs are also the behavioral cue most frequently used by researchers for species identification in the field, and some species are only detected if they are singing [4]. As a result, detection probability becomes an integral analytical adjustment for any contemporary monitoring program or population assessment strategy [5].

The species detection process (*P*) is the product of three major components (*P* = *p*_*p*_ × *p*_*a*_ × *p*_*d*_): the probability that an individual bird associated with the sample area is present (*p*_*p*_), available for detection (i.e., calling, visible, etc.) during the survey period (*p*_*a*_), and the probability it is detected by an observer given it is present and available (*p*_*d*_) [6, 7]. Availability (*p*_*a*_) is the most difficult component of the detection process to assess directly, and can have a greater effect on population estimates than other factors, including observer ability [7, 8]. Distance sampling methods [9] generate estimates of *p*_*d*_, and removal sampling [10] and time-of-detection [11] methods estimate *p*_*a*_ × *p*_*d*_. Logistic regression methods are confounded because *p*_*p*_ × *p*_*a*_ × *p*_*d*_ are inseparable though occupancy models are capable of separating *p*_*p*_ from detection probability *p*_*a*_ × *p*_*d*_ [5]. To estimate directly, factors affecting species availability for detection (*p*_*a*_), the species of interest must be present at a survey location (*p*_*p*_ = 1), and it must be detectable within a reasonable distance, which can vary by species (*p*_*d*_ = 1).

Additionally, variability in abundance during surveys can affect species detection probabilities as: *P*_*d│a*_ = 1 –(1 –*r*)^*N*^, where *N* = number of individuals in a sampling unit (abundance), and *r* is intrinsic (or individual) detection probability [12, 13]. In this case *r* is assumed to be constant because factors contributing to known heterogeneity in *r* related to individual behavior are difficult to quantify, though much attention has been paid to variables affecting species detection probability as a whole (time since sunrise, season, temperature, observer, species breeding condition etc.). Assuming *r* is constant, local abundance (*N*) will positively affect species detection probability because observers are more likely to hear a particular individual if more individuals are present and calling [12, 14, 15]. Density dependent effects such as changes in behavior in response to conspecific stimuli would cause variation in individual detection probability (*r*) and affect species detection probability, more specifically individual availability (*r*_*a*_), though this response is difficult to quantify directly [16–19].

Effects of conspecifics on bird calling are well documented in the literature. Birds are known to increase singing rates or counter singing in response to conspecifics [20–22], and practitioners have used conspecific playback to elicit bird responses during surveys [23, 24]. Additionally, birds can be attracted to new areas by using conspecific playback [25, 26]. Calling conspecifics affected per capita Golden-cheeked Warbler (*Setophaga chrysoparia*) song rates recorded with Autonomous Recording Units (ARU) and were correlated with spot-mapped densities, but direct evidence of increased singing rates would provide stronger evidence of density effects on individual availability for detection (*r*_*a*_) [22]. Similarly, heterogeneity in individual availability for detection (*r*_*a*_) can affect abundance estimation in an *N*-mixture modeling framework [13]. More specifically, density dependent changes in *r* are influenced by scenario-driven conspecific responses of individuals to other individuals calling [13].

We used data from radio-collared Northern Bobwhite (*Colinus virginianus*) breeding season surveys to determine how individual availability for detection (*r*_*a*_) is affected by intraspecific behavior and temporal covariates (minutes-since-sunrise and day-of-year). Radio-telemetry allows an observer to know unambiguously if a species is present or absent (*p*_*p*_ = 1), and a species like Northern Bobwhite has a loud and obvious call so detection probability by an observer is close to 1 (*p*_*d*_ ~1). Therefore we have the unique opportunity to isolate factors that may affect species availability (*p*_*a*_) because of known presence and assured observer detection [27]. In addition to Northern Bobwhite calls being very conspicuous and easy to detect, we chose this species because management recommendations are commonly based on results from breeding season surveys [28–30] and could have implications on the conservation of non-target species [29]. Thus variability in *r*_*a*_ of Northern Bobwhite could influence management recommendations and would impact other species [31].

Finally, Northern Bobwhite have experienced long-term range-wide population declines [32] and improvements on monitoring strategies would have important conservation implications for this species of concern. The National Bobwhite Conservation Initiative (NBCI) encourages establishment of Northern Bobwhite conservation focal areas throughout its range as a part of the Coordinated Implementation Program (CIP). The CIP uses spring breeding-season counts as a method to monitor success of habitat implementation [33]. It is important to understand how individual availability for detection (*r*_*a*_) could affect the interpretation of survey results, relating to effectiveness of habitat implementation.

Our objective was to use Northern Bobwhite breeding season surveys to examine how individual availability for detection (*r*_*a*_) is affected by conspecific cues and compare results across three common survey time lengths (3 min, 5 min, 10 min) while also accounting for time-of-day and day-of-season effects. Based on review of the literature, we hypothesized that Northern Bobwhite individual availability for detection (*r*_*a*_) would increase as the number of calling conspecifics during a count increased, would decline as minutes-since-sunrise increased, and would decline as the breeding season progressed. We also hypothesized that there would be diminishing returns of increased detection probability on observer effort and time invested for extending survey times. To our knowledge, this is the first time non-simulated data have been used to quantify individual availability for detection (*r*_*a*_).

## Materials and methods

### Study area

We conducted radio-telemetry surveys on Peabody Wildlife Management Area located in the Central Hardwoods Bird Conservation Region [34]. Peabody Wildlife Management Area is an 18,854-ha reclaimed surface mine managed by Kentucky Department of Fish and Wildlife Resources in Ohio and Muhlenberg counties, Kentucky. Kentucky Department of Fish and Wildlife Resources is the authority who issued the permission to conduct this research; Northern Bobwhite are not considered to be endangered or protected. Herbaceous cover established during reclamation was dominated by sericea lespedeza (*Lespedeza cuneata*), but also included big bluestem (*Andropogon gerardii*), little bluestem (*Schizachyrium scoparium*), Indiangrass (*Sorghastrum nutans*), and switchgrass (*Panicum virgatum*). Our focal area for surveys was a 3,321-ha unit comprised predominantly of mixed deciduous forest (22%), open herbaceous (36%), native warm-season grass (8%), and shrub (25%) cover types [35, 36].

### Radio-telemetry surveys

We used telemetry surveys conducted on Peabody Wildlife Management Area in 2010 and 2011 to document male Northern Bobwhite breeding season availability for aural detection by point count surveys. We randomly selected from a sample of >50 radio-tagged male Northern Bobwhites for location and observation, which were part of an ongoing telemetry study at Peabody Wildlife Management Area [35]. We located the observation point by homing in [37] to within approximately 50 m of the target male. We chose this distance to assure that the radio-collared bird would be detected if it called. We used signal strength and direction to estimate the distance (m) and azimuth to the hidden transmitter on the selected bird and to aid in homing [35]. Once the observation point was established, we waited 1 minute to allow for the potential disturbance of our arrival on the target individual to abate. After this acclimation period, we used a time-of-detection survey [11] by recording the calling behavior of the target (telemetry-located) radio-collared male for ten 1-minute segments. We recorded the number of times each individual radio-collared male called per minute. We also recorded all other male Northern Bobwhites within audible range (~200 m) [38] that called during the survey, and each minute within ten 1-minute segments that they called. After the 5^th^ minute, we relocated the target male to confirm the correct male was being monitored before resuming the call surveys for the remaining 5 minutes. We confirmed the final location of the target male and visually estimated the distance of the individual from the survey point when the survey was completed. We conducted surveys during all times of the day (sunrise until 17:07) from May 3–July 10, 2010 and from June 1–Aug 1, 2011. During the survey, we recorded the number of other Northern Bobwhites calling and aurally detected by the observer, the time-of-day (minutes-since-sunrise), and the date (day-of-season).

### Analyses

We assessed the effects of behavioral and temporal covariates on individual availability for detection during a 10-minute survey, number of minutes during the 10-minute survey that Northern Bobwhites called, and the number of distinct Northern Bobwhite whistles during the 10-minute survey (singing rate). Because we recorded data as minute-specific observations, we also modeled individual availability for detection for two other commonly used point-count time lengths; 3 and 5 minutes. In this case, detection probability directly estimates the probability of individual availability to call (did the individual sing or not sing during the length of the count) for the radio-collared individual during a 3-minute, 5-minute, and 10-minute survey (*r*_*a*_). We estimated breeding season individual availability for detection (*r*_*a*_) using mixed-effects logistic regression in program R [39] via package lme4 [40] for 3-minute, 5-minute, and 10-minute intervals. We knew the individual was present (*p*_*p*_ = 1) via radio telemetry and we assumed the observer would detect the individual if it called (*p*_*d*_ ~ 1) because of the proximity (50 m) of the observer to the focal bird. We modeled the number of minutes during the 10-minute survey that Northern Bobwhite called using the same package in R, but assumed a Poisson distribution to describe the number of minutes (1–10 min). Last, singing rate during the 10-minute survey was modeled assuming a zero-inflated Poisson (ZIP) distribution because we were concerned about the amount of zeros in the data, and was implemented in package pscl [41] in program R. We treated bird ID as a random effect, to account for a potential lack of independence because some individual males had multiple point counts associated with them. We were unable to fit random effects as part of the singing rate ZIP analysis because models would not converge in package pscl. We evaluated the significance of random effects using a chi-square test to compare the top mixed-effects model with and without the random effects parameter. In all instances, we used package AICcmodavg [42] for model comparison with Akaike’s Information Criterion adjusted for small sample sizes (AIC_c_).

For all analyses, we grouped surveys based on the year they were conducted (temporal), and included the number of other Northern Bobwhites calling (behavioral) at the time of the survey, minutes-since-sunrise (temporal), and day-of-season (temporal) as covariates. We considered each point count as an independent event because they were recorded on separate days for any given male. We quantified minutes-since-sunrise based on a 24-hr period, we considered May 3, 2010 as the first sampling day and as the start value of 0 for day-of-season. We did not include observer effects because we trained all observers for one week prior to allowing them to independently track via radio-telemetry and conduct point counts. Additionally, target birds were so close (~50 m) to observers that it would be very unlikely for an observer to not hear a target bird call.

We fitted a suite of 22 a priori models based on our specific objectives for evaluating availability for detection during 3-minute, 5-minute, and 10-minute surveys. We fitted a suite of 23 models to evaluate the number of minutes Northern Bobwhite called during a 10-minute survey, and we fitted a suite of 25 models to evaluate singing rate during a 10-minute survey. We included additive linear and quadratic models for each of the covariates and all combinations of the covariates. We included quadratic models because we suspected non-linear covariate relationships. We also included an interaction term for year × day-of-season because we suspected that our discrepancy in survey timing could affect individual availability for detection. We considered models with a ΔAIC_c_ ≤ 2 most competitive in explaining variability among our model set and given our data [43]. We assumed equal availability for detection among 1-minute intervals because intervals were equal in duration [10, 44]. All results are presented as means ± SE, and we used top-model mean covariate values to derive predictions from models; we did not model average. Rather than presenting complete model sets for all analyses, we only present the four most parsimonious models, the interaction model, and constant model.

We assumed population closure and no double-counting [11, 45] because radio-telemetry results suggested that Northern Bobwhite, on average, did not move significant distances (<7 m) during a 10-minute survey, and we relocated individuals immediately following each survey to ensure monitoring of the target individual. Northern Bobwhite movement during surveys was less than the recorded telemetry estimation error (9.33 ± 0.65 m) for 23 technicians [35], therefore “movement” during the survey could have been a product of human error.

## Results

In 2010, 5 observers conducted 128 point count surveys and detected 258 unmarked Northern Bobwhite males associated with monitoring 32 radio-collared males, and in 2011, 6 observers conducted 148 point count surveys and detected 358 unmarked Northern Bobwhite males associated with monitoring 34 radio-collared males during 10-min surveys. The mean number of point count surveys associated with each male was 4.37 (± 0.39). We recorded calls on 109 point counts (39.5%) during 10-min survey periods. The farthest distance a radio-collared male moved during the 10-min survey period was 60 m ($\bar{x}$ = 6.2 ± 0.61 m, *n* = 276). On average, not including the radio-collared individual, observers detected 1.5 (± 0.09, *n* = 276) Northern Bobwhite males during 3-min surveys, 1.7 (± 0.1, *n* = 276) Northern Bobwhite males during 5-min surveys, and 2.2 (± 0.1, *n* = 276) Northern Bobwhite males during 10-min surveys. Mean minutes-since-sunrise during surveys was 214 min (± 7 min) and mean day-of-season that surveys were conducted was day June 16.

**Fig 1:**
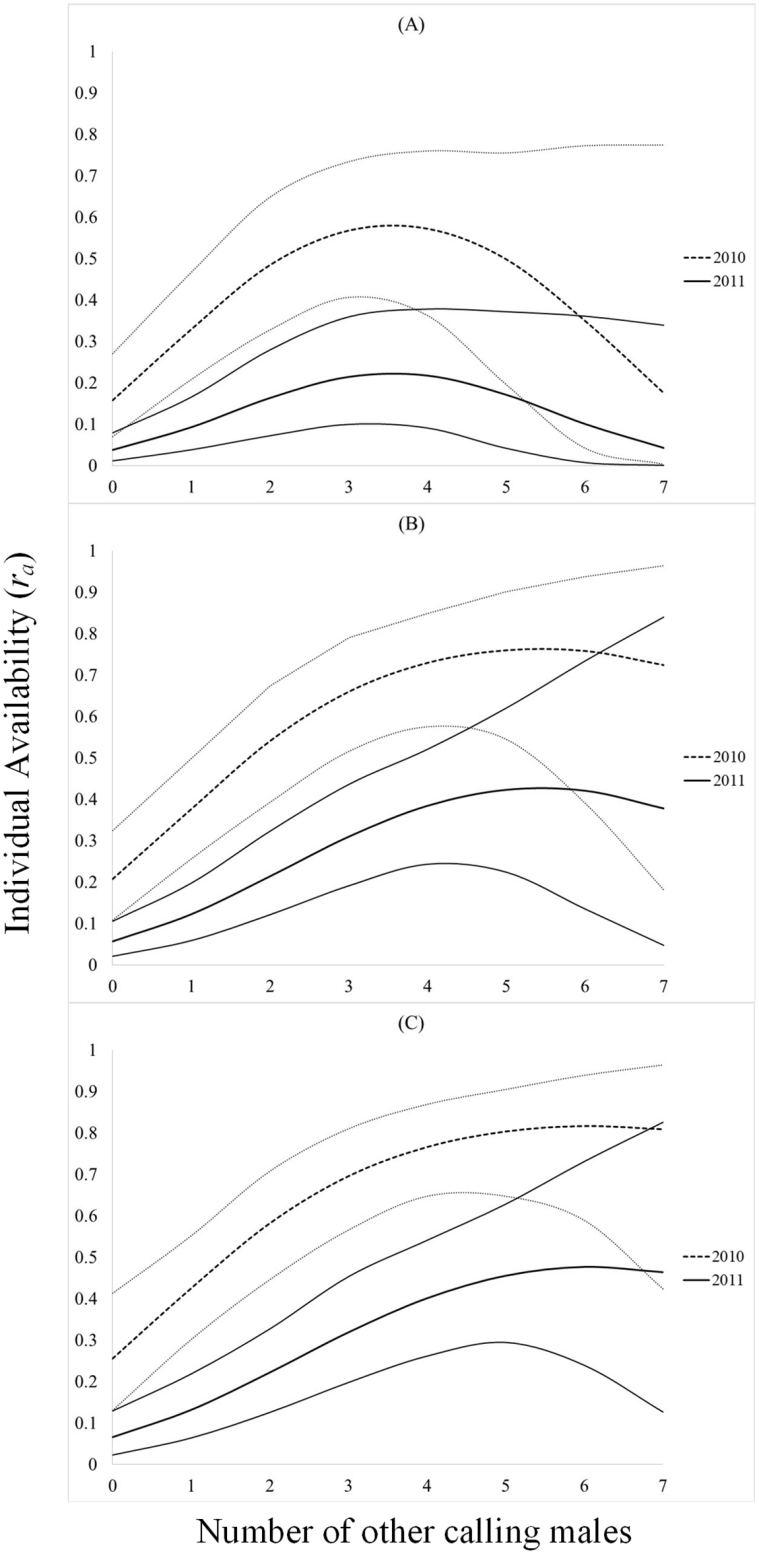
Calling conspecifics and individual availability. Effects of calling conspecifics on radio-collared male Northern Bobwhite individual availability for detection (*r*_*a*_) with 95% confidence intervals, from top models for 3-minute (A), 5-minute (B), and 10-minute (C) surveys from 2010–2011, Peabody Wildlife Management Area, KY.

**Fig 2:**
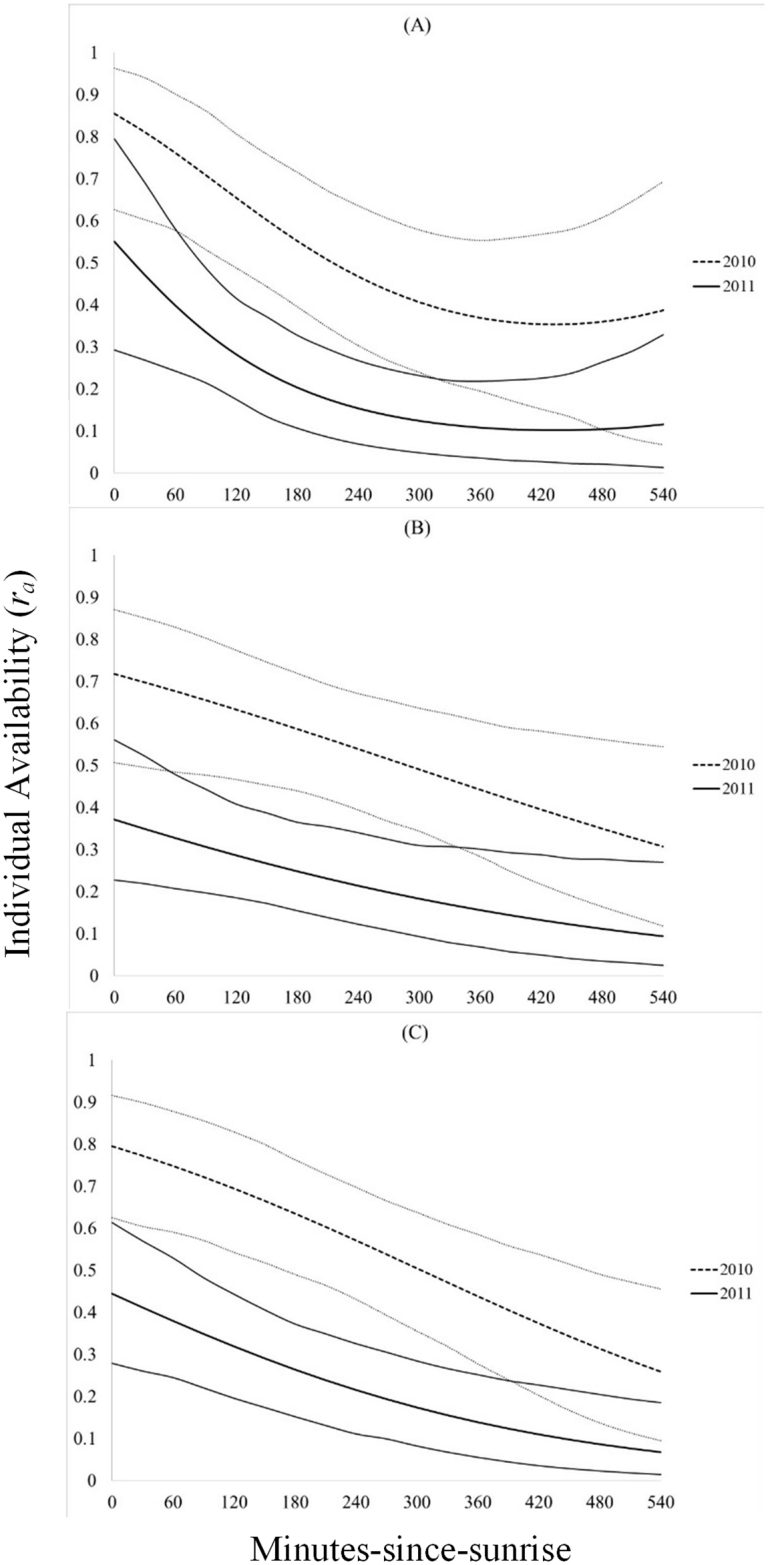
Minutes-since-sunrise and individual availability. Effects of minutes-since-sunrise on radio-collared male Northern Bobwhite individual availability for detection (*r*_*a*_) with 95% confidence intervals, from top models for 3-minute (A), 5-minute (B), and 10-minute (C) surveys from 2010–2011, Peabody Wildlife Management Area, KY.

**Fig 3:**
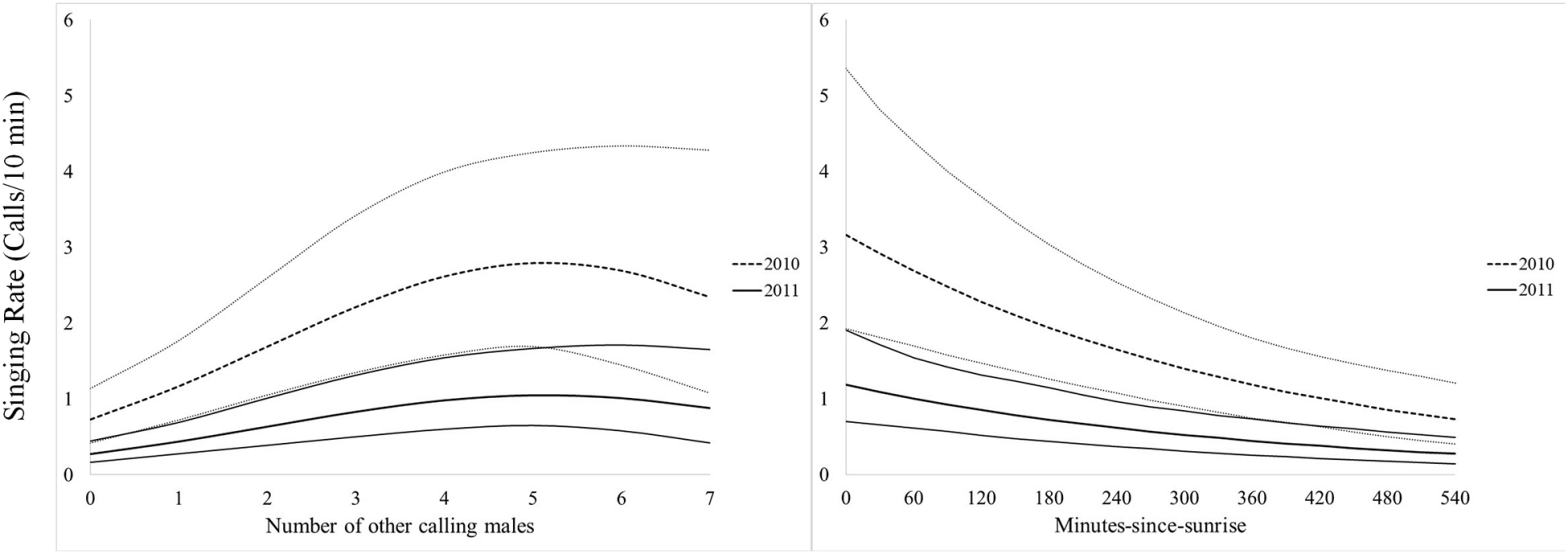
Number of minutes with a call. Effects of calling conspecifics and minutes-since-sunrise on the number of minutes with a call during a 10-minute survey with 95% confidence intervals, of radio-collared male Northern Bobwhite from 2010–2011, Peabody Wildlife Management Area, KY.

**Fig 4:**
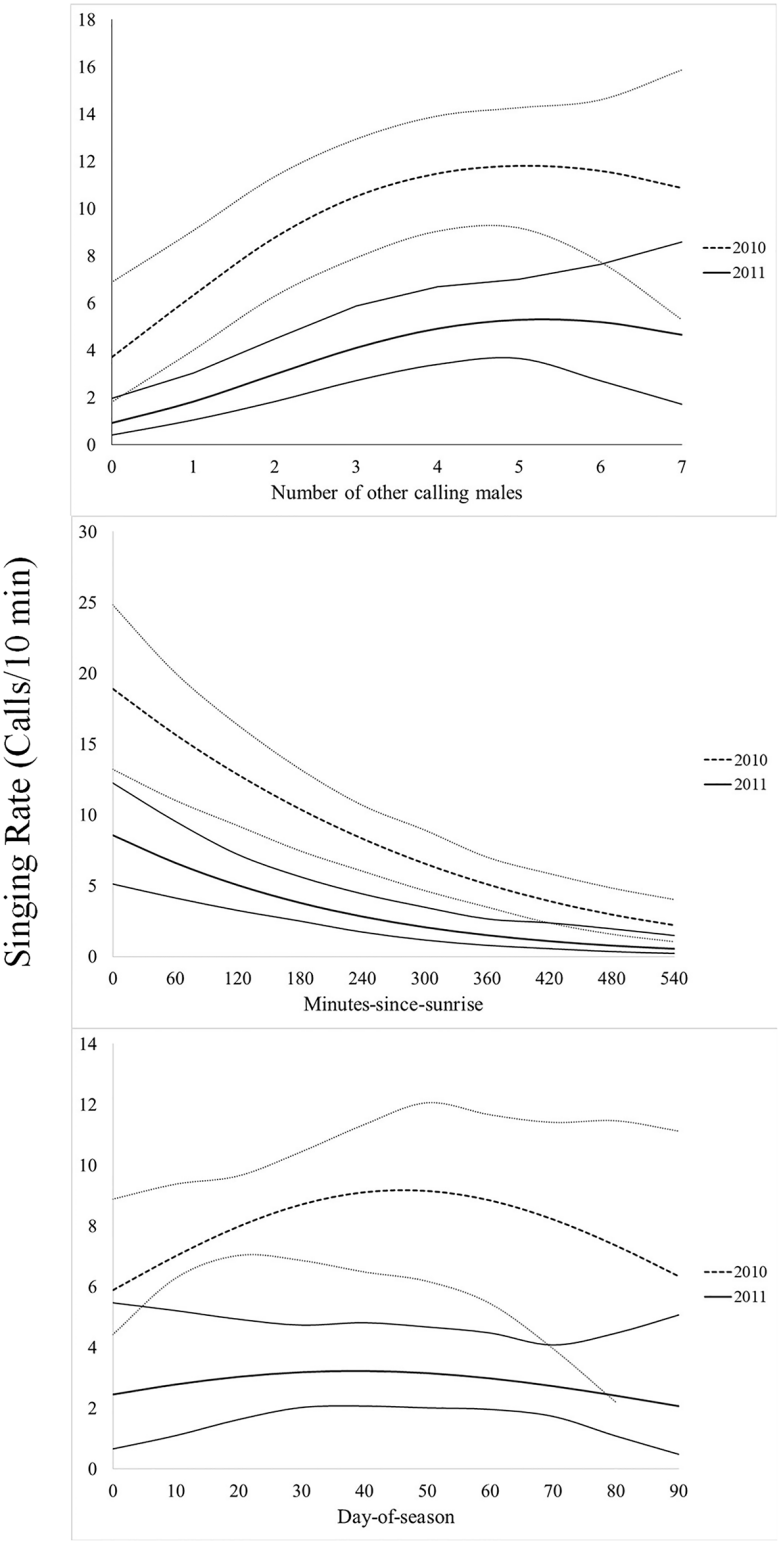
Singing rate. Effects of the number of calling conspecifics, minutes-since-sunrise, and day-of-season on the number of radio-collared male Northern Bobwhite singing rate with 95% confidence intervals, during a 10-minute survey from 2010–2011, Peabody Wildlife Management Area, KY.

In all instances, individual availability for detection (3-minute, 5-minute, and 10-minute), the number of minutes with a call during a 10-minute survey, and singing rate during a 10-minute survey was greatest in 2010, had a positive quadratic relationship to the number of other Northern Bobwhites calling (ABUN), and a negative relationship to minutes-since-sunrise (MSS; Figs 1 and 2, Tables 1–3). Random effects were only significant for the number of minutes calling models (*p <* 0.05). Day-of-season (DOS) was only retained in top models for singing rate during a 10-minute survey (Table 1). Individual availability for detection of Northern Bobwhite was greater during a 10-minute survey (0.40 ± 0.05), than during a 5-minute survey (0.31 ± 0.05), or during a 3-minute survey (0.23 ± 0.0). Individual availability for detection during a 10-minute survey was 150% greater in 2010 (61% ± 6.8) than 2011 (24% ± 5.6), and in 2011 increased by 100% when at least 1 other Northern Bobwhite called during a survey (6.5% ± 2.6 to 13.1% ± 3.7) and by 626% when at least 6 other Northern Bobwhites were calling (6.5% ± 2.6 to 47.6% ± 12.6). The magnitude of these effects was similar, albeit less substantial in 2010, and for 3-minute and 5-minute surveys (Fig 1). On average Northern Bobwhite called during 1 minute of a 10-minute survey, and called most in 2010 when at least 5 other Northern Bobwhites were calling (2.8 ± 0.66 minutes/10-min survey) and at sunrise (3.3 ± 0.88 minutes/10-min survey; Fig 3). Similarly, during a 10-minute survey in 2010, Northern Bobwhite singing rate was greater when at least 5 other Northern Bobwhites were calling (11.8 ± 1.3 calls/10 min) and at sunrise (18.9 ± 2.9 calls/10 min; Fig 4). Northern Bobwhite singing rate was also affected by day-of-season, and peaked on June 12 (5.5 ± 0.83 calls/10 min; Fig 4).

**Table 1:** Model selection results. Top competitive models of individual availability (3-minute, 5-minute, 10-minute), minutes with a call, and singing rate for radio-collared Northern Bobwhite males from 2010–2011, Peabody Wildlife Management Area, KY.

	Model^a^	ΔAIC_c_	AIC_c_ weight	Model Likelihood	No. of Parameters
3-minute	^b^Year+ABUN^2^+MSS^2^	0	0.48	1	7
	Year+ABUN^2^+MSS	0.68	0.34	0.71	6
	Year+ ABUN^2^+MSS^2^+DOS^2^	3.68	0.08	0.16	9
	Year+ABUN^2^	5.31	0.03	0.07	5
	Year+ABUN^2^+Year*DOS	9.2	0	0.01	16
	Constant	32.75	0	0	1
5-minute	^c^Year+ABUN^2^+MSS	0	0.35	1	6
	Year+ABUN^2^+MSS^2^	0.93	0.22	0.63	7
	Year+ABUN+MSS+DOS	2.59	0.10	0.27	6
	Year+ABUN^2^	2.85	0.08	0.16	5
	Year+ABUN^2^+Year*DOS	7.03	0.01	0.03	16
	Constant	40.64	0	0	1
10-minute	^d^Year+ABUN^2^+MSS	0	0.51	1	6
	Year+ABUN+MSS+DOS	1.61	0.23	0.45	6
	Year+ABUN^2^+MSS^2^	2.06	0.18	0.36	7
	Year+ABUN^2^+MSS^2^+DOS^2^	5.35	0.04	0.07	9
	Year+ABUN^2^+Year*DOS	10.62	0	0	16
	Constant	47.79	0	0	1
Minutes with a Call	^e^Year+ABUN^2^+MSS	0	0.46	1	6
	Year+ABUN^2^+MSS^2^	1.74	0.19	0.42	7
	Year+ABUN^2^+MSS^2^+DOS^2^	4.50	0.05	0.11	9
	Year+ABUN+MSS+DOS	9.00	0	0	6
	Year+ABUN^2^+Year*DOS	22.46	0	0	16
	Constant	104.88	0	0	1
Singing Rate	^f^Year+ABUN^2^+MSS+DOS^2^	0	0.49	1	14
	Year+ABUN+MSS+DOS	1.30	0.26	0.52	10
	Year+ABUN^2^+MSS^2^+DOS^2^	3.48	0.09	0.18	16
	Year+ABUN^2^+MSS	4.23	0.06	0.12	10
	Year+ABUN^2^+Year*DOS	41.16	0	0	16
	Constant	91.17	0	0	2

^a^ABUN = number of other males calling during a survey, MSS = minutes-since-sunrise, DOS = day-of-season

^b^AIC_c_ = 281.25

^c^AIC_c_ = 303.07

^d^AIC_c_ = 315.64

^e^AIC_c_ = 1182.54

^f^AIC_c_ = 1836.49

**Table 2:** Beta values and standard errors of covariates included in top competitive models of individual availability (3-minute, 5-minute, 10-minute) for Northern Bobwhite males from 2010–2011, Peabody Wildlife Management Area, KY.

Model Variable^a^	3-minute^b^	SE	z	p-value	5-minute	SE	z	p-value	10-minute	SE	z	p-value
Intercept	-1.48	0.63	-2.34	<0.05	-2.11	0.5	-4.22	<0.01	-1.7	0.49	-3.47	<0.01
YEAR	1.57	0.43	3.73	<0.01	1.46	0.39	3.45	<0.01	1.58	0.4	3.30	<0.01
ABUN	11.27	3.02	-2.50	<0.05	9.24	2.68	-1.64	0.10	8.36	2.53	-1.58	0.11
ABUN^2^	-15.84	6.33	-2.51	<0.05	-8.48	5.17	-2.17	<0.05	-6.82	4.31	-2.97	<0.01
MSS	-11	4.39	1.75	0.08	-3.24	1.49	3.77	<0.01	-4.46	1.5	3.97	<0.01
MSS^2^	12.7	7.25	3.68	<0.01	NA	NA	NA	NA	NA	NA	NA	NA
DOS	NA^c^	NA	NA	NA	NA	NA	NA	NA	NA	NA	NA	NA
DOS^2^	NA	NA	NA	NA	NA	NA	NA	NA	NA	NA	NA	NA
RandomEffects^d^	0.48	0.69	2.62	0.11	0.35	0.6	1.56	0.21	0.5	0.7	3.43	0.06

^a^ABUN = number of other males calling during a survey, MSS = minutes-since-sunrise, DOS = day-of-season

^b^Singing Rate Zero-inflated Poisson beta values are not reported, which is why there is a discrepancy with Table 1 parameters

^c^NA = parameter not included in the top model

^d^RandomEffects = SE is actually Standard Deviation, z test statistic is actually chi-square test statistic

**Table 3:** Beta values and standard errors of covariates included in top competitive models for minutes with a call, and singing rate for radio-collared Northern Bobwhite males from 2010–2011, Peabody Wildlife Management Area, KY.

Model Variable^a^	Minutes with a call	SE	z	p-value	Singing Rate^b^	SE	z	p-value
Intercept	-0.72	0.29	-2.53	<0.05	2.23	0.18	12.58	<0.01
YEAR	0.98	0.32	5.30	<0.01	0.31	0.07	0.34	0.73
ABUN	5.25	0.99	-3.24	<0.01	0.19	0.56	-0.41	0.68
ABUN^2^	-5.1	1.58	-4.70	<0.01	-0.34	0.84	-6.45	<0.01
MSS	-2.72	0.58	3.04	<0.01	-2.09	0.32	2.89	<0.01
MSS^2^	NA^c^	NA	NA	NA	NA	NA	NA	NA
DOS	NA	NA	NA	NA	2.46	0.85	-2.48	<0.05
DOS^2^	NA	NA	NA	NA	-2.27	0.92	4.25	<0.01
RandomEffects^d^	1.2	1.1	159.3	<0.01	NA	NA	NA	NA

^a^ABUN = number of other males calling during a survey, MSS = minutes-since-sunrise, DOS = day-of-season

^b^Singing Rate Zero-inflated Poisson beta values are not reported, which is why there is a discrepancy with Table 1 parameters

^c^NA = parameter not included in the top model

^d^RandomEffects = SE is actually Standard Deviation, z test statistic is actually chi-square test statistic

## Discussion

We defined a new parameter, individual availability for detection (*r*_*a*_), and used field-collected data to relate the effects of calling conspecifics to breeding individual availability for detection for a bird species, the Northern Bobwhite. The activity of calling conspecifics was correlated with individual availability for detection during a 10-minute count and varied significantly, ranging from <7% in 2011 in the absence of conspecific stimuli, to >80% in 2010 if 6 other Northern Bobwhites were calling during a survey (Fig 1). The number of Northern Bobwhite calls was also significantly positively affected by stimuli of conspecifics, which was likely the greatest contributing factor to the increase in individual availability for detection. Not only were Northern Bobwhite more likely to call at least once during a 10-minute count as a result of conspecific stimuli, but they were also calling during more minutes, and at a greater rate (Figs 3 and 4). Though we did not account for some environmental factors (temperature, barometric pressure, wind speed, predator density, etc.), or behavioral factors (pairing status, nesting status, etc.) we included temporal variables (time-of-day and day-of-season) in analyses, which typically have a greater effect on avian detection probability. Because our results are correlative, we recognize that other unforeseen or unmeasured variables could be causing these results to be spurious. Future studies could use playback in conjunction with radio-collared birds to experimentally affirm our results, and include additional variables.

Our Northern Bobwhite availability for detection during a 10-minute count (0.40) was less than that derived from other researchers using field data (~0.9) [7]. The reason for this discrepancy is likely because we directly estimated individual availability for detection through real-time telemetry-based calling surveys conducted on a large sample of individual males, across two years, while accounting for temporal and behavioral factors. Previously, a combination of double-observer and time-to-detection methodologies was used to quantitatively derive species availability estimates, rather than directly quantify individual availability for detection [7].

The concept of individual heterogeneity affecting availability for individual capture or individual detection has always complicated species population modeling [12, 46, 47]. Variability in individual song rate for birds is well documented, specifically relating to breeding condition, time-of-day, or day-of-season [21, 48]. These extrinsic factors are easily accounted for as part of the species detection process through inclusion of covariates. Intrinsic (individual) heterogeneity as part of the detection process is more difficult to account for, but can be indirectly modeled in *N*-mixture and occupancy models [12, 47]. If individual availability for detection is high (>0.5), or heterogeneity in individual availability for detection is random, then parameter estimates remain unbiased. However, if individual availability for detection is low (<0.2), non-random or directional, then it can produce biased parameter estimates [47]. When there is a positive density-dependent relationship on individual availability for detection, then estimated abundance will be biased low as a result of low individual availability, i.e. fewer individuals are singing and thus less likely to be detected during surveys.

Many species of birds respond positively to auditory cues of conspecifics [16, 49, 50]. This is especially true for bird species that use visual and auditory cues from conspecifics as indicators for mate selection and reproduction [51, 52]. Some species use cues from conspecifics as alarm behaviors in the presence of a threat or predator, to locate food or water resources, or to assess habitat quality [24, 53, 54]. These changes in behavior in response to conspecifics can be problematic in experimental survey designs, specifically assessments of species spatial distributions and species populations. For instance, low density areas of Golden-cheeked Warblers had lower detection probabilities [22]. Similarly, counter-singing rates of Black-throated Blue Warblers (*Setophaga caerulescens*) were lower in experimentally reduced density areas [55]. Male Eurasian Eagle Owls (*Bubo bubo*) used conspecific cues to dictate calling rate, and in low-density areas male calling rates were reduced [20].

Although Northern Bobwhite do not defend distinct territories per se, other researchers have commented on conspecifics positively affecting calling behavior in the fall and during the breeding season [56, 57]. In the fall, Northern Bobwhite use covey calls to maintain contact with surrounding coveys, and the rate of these calls is positively related to the number of other coveys calling [57]. Female Northern Bobwhite playback can elicit breeding male calling responses, but these responses are confounded by the uncontrolled presence of other males calling during the playback and impractical for non-playback surveys because females rarely vocalize [56]. Similarly, playback recordings of male Northern Bobwhite breeding vocalizations elicited increased calling rates [17], but results were confounded by the lack of experimental control for surrounding “real” males calling so the reported response could be misinterpreted as a result of playback rather than a response to other males. Also, individuals were not marked and could not be identified, so analyses could not produce estimates of individual availability for detection. Playback has been used to increase detection probabilities for a number of bird species [16–19, 58, 59], but all of these studies have similar practical limitations on surveys where individuals are unknown and the design is not paired with controlled populations.

Annual variability in species populations is accepted as inherent in population parameters of interest (abundance, density, population size), and often accounted for through a detection probability. Unaccounted for variability in the observation processes, however, could bias estimates of annual change. We documented significant annual differences in individual availability for detection, minutes during which a Northern Bobwhite called, and singing rate of Northern Bobwhite. We can only speculate about why calling behavior changed so dramatically between years but the ramifications of this result are substantial. We hypothesize that the observed dramatic decline in individual availability for detection during 2011 was in response to noise created by a 13-year brood (Brood XIX) cicada (*Maigcicada* spp.) emergence which occurred during that year in west-central Kentucky [60]. Northern Bobwhite individuals could have been responding to the increased background noise associated with the cicadas and singing less [61], or individuals could have been exploiting an abundant food resource foraging for the cicadas, and spent less time singing [62]. We did not collect any formal data about cicada abundance influencing background noise levels, so our hypothesis is purely speculative and requires further examination.

Regardless of the cause for annual differences, the magnitude of this difference is larger than we expected. Implications are that actual changes in Northern Bobwhite populations over time could easily be masked by the annual variability in individual calling behavior. For instance, based on mean minutes-since-sunrise and mean number of other calling males, Northern Bobwhite individual availability for detection during a 10-minute survey was 61% in 2010 compared to 24% in 2011. This simple effect would translate into an apparent adjusted population size that is approximately 150% greater in 2011 than 2010. This difference in population size is only a reflection of a behavioral change, and does not necessarily reflect any changes in the actual underlying populations.

## Conclusions

In recent years, Northern Bobwhite conservation has focused on identifying large areas of usable space where recovery efforts will be the most effective [63, 64]. These recovery efforts use data from breeding-season aural surveys to determine Northern Bobwhite densities or abundances, which are then used as a metric of restoration project success [65]. However, Northern Bobwhite in recovery areas with low populations will not be stimulated by other calling males, are less likely to call, call less frequently, and call fewer times per minute, reducing their individual availability and likelihood to be counted on a survey, biasing population estimates low if *r*_*a*_ is not accounted for.

We recognize that the effects of conspecifics on individual availability for detection of Northern Bobwhite are not easily incorporated into routine point-count survey monitoring for many managers. Because Northern Bobwhite recovery projects rely on population density or abundance estimates as a metric of success, it may be necessary to account for this in the modeling process. One option for managers using breeding-season counts to assess population recovery or management effectiveness is to include an estimate of *r*_*a*_ (Fig 1) in *P*_*d│a*_ = 1 –(1 –*r*_*a*_)^*N*^ which corresponds to one of our commonly used survey times (3-min, 5-min, or 10-min). Alternatively, those tracking populations using estimates derived from *N*-mixture or occupancy models could account for positive density-dependent effects on *r*_*a*_ by including our quadratic function in *P*_*d│a*_ = 1 –(1 –*r*_*a*_)^*N*^ while estimating detection probabilities. Our results also suggest that another option for increasing Northern Bobwhite availability for detection is to extend survey times, though based on objectives, it may be better to conduct more surveys rather than longer ones. These conspecific effects likely affect other taxa, in particular birds. Additional studies using radio telemetry can be used to explicitly document the magnitude of effects [8], and then practitioners can weigh their importance.

## Supporting information

S1 Dataset Radio-collared Northern Bobwhite data. These are raw detection histories and associated variables from radio-collared Northern Bobwhite individuals used for all analyses.
(XLSX)
